# Automatic morphometry in Alzheimer's disease and mild cognitive impairment^☆^^☆☆^

**DOI:** 10.1016/j.neuroimage.2011.03.014

**Published:** 2011-06-15

**Authors:** Rolf A. Heckemann, Shiva Keihaninejad, Paul Aljabar, Katherine R. Gray, Casper Nielsen, Daniel Rueckert, Joseph V. Hajnal, Alexander Hammers, The Alzheimer's Disease Neuroimaging Initiative

**Affiliations:** a The Neurodis Foundation (Fondation Neurodis), Lyon, France; b Centre for Neuroscience, Division of Experimental Medicine, Department of Medicine, Imperial College, London, UK; c Dementia Research Centre, UCL Institute of Neurology, London, UK; d Department of Computing, Imperial College, London, UK; e Imaging Sciences Department, MRC Clinical Science Centre, Imperial College, London, UK

**Keywords:** Image segmentation, Brain pathology, Brain atlas, Magnetic resonance imaging, Alzheimer's disease, Mild cognitive impairment

## Abstract

This paper presents a novel, publicly available repository of anatomically segmented brain images of healthy subjects as well as patients with mild cognitive impairment and Alzheimer's disease. The underlying magnetic resonance images have been obtained from the Alzheimer's Disease Neuroimaging Initiative (ADNI) database. T1-weighted screening and baseline images (1.5 T and 3 T) have been processed with the multi-atlas based MAPER procedure, resulting in labels for 83 regions covering the whole brain in 816 subjects. Selected segmentations were subjected to visual assessment. The segmentations are self-consistent, as evidenced by strong agreement between segmentations of paired images acquired at different field strengths (Jaccard coefficient: 0.802 ± 0.0146). Morphometric comparisons between diagnostic groups (normal; stable mild cognitive impairment; mild cognitive impairment with progression to Alzheimer's disease; Alzheimer's disease) showed highly significant group differences for individual regions, the majority of which were located in the temporal lobe. Additionally, significant effects were seen in the parietal lobe. Increased left/right asymmetry was found in posterior cortical regions. An automatically derived white-matter hypointensities index was found to be a suitable means of quantifying white-matter disease. This repository of segmentations is a potentially valuable resource to researchers working with ADNI data.

## Research highlights

► Free repository of MR-based anatomical brain image segmentations. ► Based on ADNI, covering normals and patients. ► Volumetric analysis corroborates previous findings. ► Asymmetry measures correlating with disease progression. ► Automatically derived quantitative index of white-matter disease.

## Introduction

This paper presents results of a project that aims to provide anatomical labels based on automatic segmentation for magnetic resonance (MR) brain imaging data supplied by the Alzheimer's Disease Neuroimaging Initiative (ADNI). The result of this work is made available to the general scientific community via the same channels as the source ADNI data.

Anatomical segmentations of structural images of the human brain can be used for a plethora of purposes. A principal motivation is to understand the impact of neurodegeneration, trauma, epilepsy and other conditions on the brain's macroscopic structure. Such understanding leads to morphometric descriptors with the potential to serve as biomarkers for the diagnosis and monitoring of brain disease (Colliot et al., 2008; Duchesne et al., 2008; Heckemann et al., 2008; Klöppel et al., 2008). Beyond the realm of morphometric analysis, individual anatomical segmentation is frequently used in the analysis of functional imaging data, e.g. to precisely locate areas of hypo- or hypermetabolism within the subject's own anatomical reference frame. Anatomical segmentation also enables studies of regional connectivity based on diffusion tensor imaging [e.g. Traynor et al. (2010)].

ADNI MR imaging data have hitherto been provided with only minimal amounts of segmentation information. For a subset of ADNI images, labels of the left and right hippocampi are available. These labels have been generated using a semiautomatic tool (SNT, Medtronic Surgical Navigation Technologies, Louisville, CO) that relies on manual seed point placement. In work by Hsu et al. (2002), the SNT tool was claimed to yield hippocampal volume measurements equivalent to a manual delineation protocol, but the validation was not entirely convincing: Hsu et al. make reference to previous work by Watson et al. (1992), but the protocol described there finds distinctly larger volumes in normal adult hippocampi (Watson — right: 5264 ± 652 mm^3^, left: 4903 ± 684 mm^3^; Hsu — right: 3103 ± 505 mm^3^, left: 2945 ± 503 mm^3^). Furthermore, the SNT method yields volume measurements that are yet smaller than those of the manual reference (right: 2323 ± 326 mm^3^, left: 2275 ± 253 mm^3^). Both the validation and anatomical coverage of available ADNI segmentation data are thus limited.

Beyond the hippocampus, researchers requiring anatomical labels of ADNI data have three choices:

1. Normalize subject images to a reference space and apply one of a choice of anatomical volume or surface atlases available for this space [e.g. Talairach (Talairach and Tournoux, 1988), AAL (Tzourio-Mazoyer, 2002), Maximum Probability Brain Atlas (Hammers et al., 2003), The Whole Brain Atlas,^3^ LPBA40 (Shattuck et al., 2008), PALS-B12 (Van Essen, 2005), the Freesurfer atlas Fischl et al. (2004), or a purpose-made atlas]. This can be a simple solution, in particular if other parts of the analysis already require spatial normalization. Since the segmentation process takes place in the common space, an inverse normalization has to be carried out in order to recover the volume and shape of segmented regions in native space. This approach is typically based on a single-subject atlas or maximum-probability atlas. The latter are generally preferable because they tend to eliminate idiosyncrasies due to anatomical variants in individual subjects. Success depends on the suitability of the chosen atlas, as well as the suitability and robustness of the chosen spatial normalization algorithm.
2. Carry out anatomical segmentation according to an existing or tailored protocol for manual region outlining in individual subject space. A full outlining protocol has been described by Hammers et al. (2003); other examples include the protocol by Shattuck et al. (2008) and another by Filipek et al. (1989). A further protocol for cortical labeling is under development as a collaborative project (brainCOLOR^4^). These methods require training of an operator in the chosen protocol and are expensive in terms of operator time and validation requirements, with costs rising approximately linearly with the number of images to be segmented and the number of regions labeled. The resulting segmentations are subject to intraobserver and interobserver variation.
3. Use one of a choice of semiautomatic approaches that require manual input, such as landmarks or seed points. Examples are SNT as noted above, Cardviews [Center for Morphometric Analysis, Massachusetts General Hospital, Boston, MA, USA, (Rademacher et al., 1992)], CARET [cortex only, Washington University School of Medicine, Saint Louis, MO, USA (Van Essen, 2005)] and LDDMM (Beg et al., 2004; Csernansky et al., 2004). Compared to manual outlining, interobserver variation is reduced, since even if the manual input varies within a certain range, the algorithms tend to arrive at the same results. These approaches are less labor-intensive, but the costs are still closely tied to the number of target images and regions.
4. Carry out anatomical segmentation in individual space using a fully automatic procedure. Software packages are available that implement the required functionality, but have limitations. For example, Mindboggle (Klein and Hirsch, 2005) and its extension using multiple atlases (Klein et al., 2005) are designed for cortical segmentation only, while the FS + LDDMM method achieves limited accuracy (Khan et al., 2008). Such approaches typically place a high demand on the computing infrastructure. An exception in this respect is the work by (Lötjönen et al., 2010), which is designed to reduce the computational demand sufficiently to make multi-atlas segmentation clinically feasible.

The present work is an instance of the fourth option. We have generated anatomical labels for ADNI MR images and provide them for download along with other ADNI data (http://www.loni.ucla.edu/ADNI). We present segmentations of 816 subjects' screening and baseline images into 83 regions, along with a statistical description of regional volumes.

To obtain automatic segmentations, we used multi-atlas propagation with enhanced registration [MAPER, Heckemann et al. (2010)]. This is a refined version of a previously validated approach (Heckemann et al., 2006). MAPER is the first automatic whole-brain multi-region segmentation method that has been shown to yield robust results in subjects with neurodegenerative disease. It uses training data (“atlases,” images with reference segmentations) to segment T1-weighted brain MR images of any provenance into anatomical regions (Heckemann et al., 2010). We showed in previous work (Heckemann et al., 2006) that the accuracy achieved with MAPER is only slightly inferior to that of manual segmentation performed by a trained operator, and that the procedure is robust in the face of anatomical variation in the target subjects, specifically ventricular enlargement as seen in aging and neurodegeneration.

The implementation of MAPER used here relies on software tools sourced from the Image Registration Toolkit [IRTK,^5^ Rueckert et al. (1999)] and from Nifty Reg,^6^ (Modat et al., 2010). In a comparison of tools for intersubject registration of MR brain images, IRTK was recently found to be among the best-performing ones (Klein et al., 2009). Two other tools [SyN^7^ (Avants et al., 2008) and ART^8^] (Ardekani et al., 2005) achieved more consistent results than IRTK in the comparison by Klein et al. Nevertheless, when working with heterogeneous data, we found IRTK to be more robust than ART and SyN, in particular when source (atlas) MR images had been acquired on different scanners than the target data for segmentation [e.g. ADNI images, Heckemann et al. (2010)]. ART and SyN have been shown to be suitable for registering pairs of images of identical provenance. MAPER is characterized by its robustness towards ventricular distension in the target subject. To achieve this, it relies on IRTK's ability to register multi-spectral tissue probability maps using cross correlation as the similarity measure, a feature that, to our knowledge, has not been implemented elsewhere. Our choice of IRTK rests on these two factors – robustness towards both intensity differences and typical pathology – although MAPER could in principle be implemented using other toolkits.

We validate the results using the volumes of the segmented regions as well as agreement measures between segmentations of images that have been serially acquired at different field strengths, and document limitations of the automatic procedure and the generated results for the benefit of future users of the data. We found that signal changes caused by white-matter disease can result in misclassification of tissues and lead to distortions in the segmentations. To quantify this influence, we describe and validate an automatically generated index. Finally, we show that statistical analyses of the automatically generated segmentations confirm previous observations of morphometric changes in Alzheimer's disease and mild cognitive impairment.

## Materials and methods

### MR data

Atlas data as required for MAPER consisted of 30 T1-weighted 3D image volumes acquired from healthy young adult volunteers at the National Society for Epilepsy at Chalfont, UK. Details of the acquisition are in Hammers et al. (2003). Hand-drawn segmentations of 83 structures had been previously prepared according to the protocols described in Hammers et al. (2003) and Gousias et al. (2008). Segmentation protocols are also available at http://www.brain-development.org.

MR images of patients with Alzheimer's disease and mild cognitive impairment as well as healthy elderly subjects were obtained from the ADNI database (www.loni.ucla.edu/ADNI).^9^ The research presented here aligns with the primary goal of ADNI, which has been to test whether serial magnetic resonance imaging (MRI), positron emission tomography (PET), other biological markers, and clinical and neuropsychological assessment can be combined to measure the progression of mild cognitive impairment (MCI) and early Alzheimer's disease. The full repository of ADNI images was accessed in February 2010. The clinical information was retrieved in August 2010. Each subject was assigned to one of five diagnosis groups: healthy subjects (HS), mild cognitive impairment with no conversion within the observation period (stable MCI, s-MCI), mild cognitive impairment at baseline, with progress to Alzheimer's disease within the observation period (p-MCI), Alzheimer's disease (AD), and Other (O). The latter assignment was used as a “catch-all” for subjects who did not fit the other categories, for example if ADNI noted a reversion from AD to MCI. The observation period was 24 11 months.

### Preprocessing

As envisaged in the ADNI study design, images were obtained from the ADNI database in fully preprocessed versions. Depending on the scanner source, preprocessing included all or some of *GradWarp* geometric distortion correction (Jovicich et al., 2006), *B1 nonuniformity correction* to compensate for signal inhomogeneity (Jack et al., 2008), *N3* bias field correction (Sled et al., 1998) and *phantom scaling*. We chose the originally supplied linearly scaled images, irrespective of problems reported on a subset,^10^ as linear scaling issues do not affect the segmentation procedure. Likewise, volume measurements, once normalized by intracranial volume as measured on the same source image, are unaffected by linear scaling.

To match the requirements of the MAPER procedure, we applied further preprocessing for brain extraction and tissue classification, as described in the following. Utilities used for these steps were taken from the Image Registration Toolkit [IRTK, Rueckert et al. (1999)], from the FSL suite (Smith et al., 2004) and from the ANTs toolkit (Avants et al., 2010).

For the brain extraction step, binary masks covering both intracranial white matter and gray matter (WM + GM) were available as the starting point. These had been generated as part of an earlier project using MIDAS, a semi-automatic procedure described elsewhere (Freeborough et al., 1997). Each mask was extended to cover the intracranial region generously by blurring (6 mm Gaussian kernel), thresholding at 27% and hole-filling. FSL FAST was applied to identify cerebrospinal fluid (CSF) within the pre-masked region. The original WM + GM mask was extended by the resulting CSF mask to obtain a complete intracranial mask that excluded meninges, sinuses and extracranial tissue. The original, semi-automatically created WM + GM mask is fully contained within the intracranial mask, reducing the impact of operator-dependent variability on the intracranial volume measurement.

**Fig. 1:**
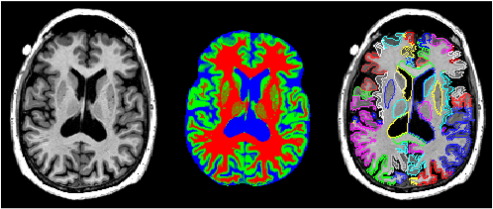
Transverse section through the image of a healthy subject (73 year-old male, image ID I90026). Left panel: T1-weighted MR image. Middle panel: result of tissue classification with FAST; probability maps for each tissue class are combined into a single multi-spectral volume. Right panel: segmentation generated with MAPER. To create an accurate impression of the original resolution, the figure has been rendered without interpolation.

Individual tissue probability maps for CSF, GM and WM obtained using FSL FAST were combined into a single multi-spectral volume. Fig. 1 shows a sample section from an image of a healthy subject. A binary maximum-probability gray matter mask was extracted from the discrete tissue class image generated by FAST.

**Fig. 2:**
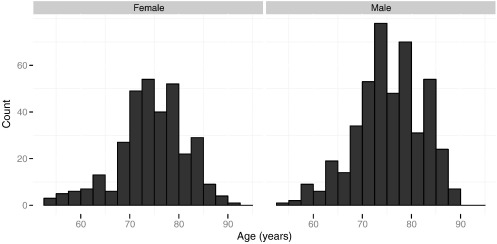
Age distribution of included subjects.

T1-weighted screening (1.5 T) and baseline (3 T) images from the ADNI repository were obtained for all subjects for whom MIDAS-prepared brain masks were available. After removing data sets that had been withdrawn by ADNI after the download, a total of 996 images on 816 subjects (1.5 T: 811, 3 T: 185; of which paired: 180) were segmented and quality assessments carried out (cf. Statistical and visual analysis). For statistical analysis, only subjects in the HS, s-MCI, p-MCI, and AD groups (i.e. excluding the “Other” category), and of these only those who passed the outlier analysis (Outlier analysis section) were included (777 subjects, 953 images, 1.5 T: 772; 3 T: 181, of which paired: 176). The age distribution of included subjects is shown in Fig. 2. A breakdown by diagnostic group and gender is given in Table 1.

In the 176 included subjects for whom images had been acquired at both field strengths, the 3 T image was typically acquired within weeks after the 1.5 T image (median 22 days, range 0–112 days).

### Segmentation

The MAPER procedure for robust, automatic segmentation of T1-weighted MR images of the human brain has been described and validated previously (Heckemann et al., 2010). Each target is paired with each of the atlases to generate an individual atlas-based segmentation. The steps are summarized in Table 2. In Steps 3 and 4, alignment of details in the image pair was achieved by optimizing a free-form deformation (FFD) represented by displacements on a grid of control points blended using cubic B-splines (Rueckert et al., 1999). These steps are carried out using each of the 30 atlases in turn, resulting in 30 segmentations, which are subsequently combined using vote-rule decision fusion (Rohlfing et al., 2004; Kittler et al., 1998).

In contrast to the approach discussed in Heckemann et al. (2010), where the entire registration was done in IRTK, we used Nifty Reg Version 1.3 (Modat et al., 2010) to carry out the detail-level registration (Step 4). The transformed and interpolated output from IRTK was used as the starting point. Nifty Reg is a particularly efficient implementation of the same FFD registration. To compare the accuracy of MAPER based on the combination (IRTK and Nifty Reg) with MAPER based on pure IRTK, we carried out a leave-one-out cross-comparison on the 30 atlas sets with both implementations, following the method described in Heckemann et al. (2010). Agreement between the generated and the manual label sets was measured using the mean Jaccard coefficient [*JC*; intersection divided by union (Jaccard, 1901)] across all 83 regions. The mean *JC*_*m*_ across the 30 atlas images was 0.691 for both methods [range 0.653–0.714, SD 0.0141 (IRTK), range 0.664–0.711, SD 0.0134 (IRTK and Nifty Reg)].

### Quantifying white-matter disease

White-matter disease (WMD), characterized by diffusely hypointense regions within the white matter, is frequently seen in elderly subjects, and specifically in those with dementia (Black et al., 2009). Such regions can adversely affect the functioning of intensity-based methods. In the case of FSL FAST, they tend to be incorrectly labeled as gray matter, and this can impact subsequent processing — in the case of MAPER, resulting segmentations can be distorted. In particular, the lateral ventricle, the caudate nucleus, and the insula can be overestimated (cf. Outlier analysis). We developed a procedure to estimate the amount of white matter that is misclassified. The estimate is derived from a set of different label images derived from the T1-weighted image:

• A binary WM segmentation of the target by majority vote fusion of transformed atlas WM segmentations (each estimated from their intensities by FAST; note the atlas subjects are healthy young adults not affected by WMD): *M*_*W*_
• A binary GM segmentation of the target generated with FAST: *F*_*G*_
• A semi-automatically generated binary label covering white matter and gray matter of the target, as described in Preprocessing section: *S*_*B*_
• A binary segmentation of both lateral ventricles in the target extracted from the fusion of the transformed atlas labels: *M*_*V*_

An image with suspected WMD voxels is generated as

W=A∪B

where

$$A=(M_{W}\cap F_{G})⊖E$$

$$B=(M_{V}\cap S_{B})⊖E$$

and E is a 3 3 3 structuring element used for eroding the intermediate images (this operation symbolized by ⊖).

In subjects where WMD leads to hypointensities that coincide with white matter, as identified by transforming atlas WM segmentations, such regions will be labeled by the intermediate Image *A*. In subjects where hypointensities border on the lateral ventricles, Image *B* will capture the affected regions. The volume of the resulting label *W*, normalized by the intracranial volume, provides an indication of the subject's WMD load. In the following, we refer to this measure as the white-matter hypointensities index (WMHI).

We assessed the validity of the WMHI by comparison with a semiquantitative rating. We adapted the rating scale described by Wahlund et al. (2001), which was designed for X-ray computed tomography and T2-weighted MR images, for use with T1-weighted images:

0: No hypointensities clearly identifiable as lesions
1: Focal lesions
2: Beginning confluence of lesions
3: Diffuse involvement of the entire region

The WMHI distribution was highly nonlinear with a small number of high values. To select a subset for visual scoring, we ranked the images according to WMHI, divided the sample into three equal parts, and sampled in a proportion of 42:21:7 from each group, yielding a total of 70 images for review.^11^ An experienced rater (AH) who was blinded to WMHI, age, and diagnosis assigned the score after reviewing the T1-weighted images in three orthogonal planes. Where subjectively appropriate, based on comparisons within the sample, the rater assigned a tendency to the score, which was recorded as an addition or subtraction of 0.3 points to or from the integer score.

### Masking based on tissue class

Depending on the application, it may be desirable to use segmented regions that have been multiplied with a binary tissue class label. In particular, since aging and Alzheimer's disease are characterized by cortical neuronal loss, the GM portion within each cortical label is often more relevant than the full label containing both GM and WM. We thus provide both raw segmentation data and masked versions. For the latter, regions with a substantial GM portion have been masked with a GM label (all except ventricles, central structures, cerebellum and brainstem), and the lateral ventricles have been masked with a CSF label. Unless otherwise noted, the analysis results reported in this work are based on the masked label sets.

### Statistical and visual analysis

We assembled and analyzed the results of volumetry on all structures in all target subjects using standard statistical methods as provided by the R environment (http://www.r-project.org/).

Segmentation failures typically lead to grossly inaccurate estimations of the volume of individual regions. To detect outliers in the data, we grouped the images by diagnosis, gender and field strength, and determined per-group means and standard deviations of the regional volume (normalized by intracranial volume; masked by GM except for ventricles, central structures, brainstem and cerebellum). On this basis, all region volumes were converted to *z* scores. Regions where the *z* score was greater than 4 or less than − 4 were flagged as outliers. Images containing outlier regions were visually assessed by an experienced reader (RAH). Label outlines were superimposed on the MRI image and the flagged region and its neighborhood viewed in the transverse, sagittal and coronal planes. The segmentation quality was rated on a visual analog scale from 1 to 5 (1: failed segmentation; 2: poor boundary matching, but correct indication of the relative position of neighboring regions; 3: fair; 4: good segmentation with minor boundary mismatches, 5: excellent segmentation with exact boundary matching). The likely cause of the outlying size of the region, based on the reader's subjective impression, was identified and recorded. The remainder of the image was searched in the transverse plane for obvious label mismatches beyond the flagged region and a note of the overall impression recorded.

Statistical analysis was carried out with a view to comparing diagnostic groups and determining potential volumetric criteria characteristic for Alzheimer's disease or impending progression from mild cognitive impairment. We also used MAPER measurements to determine balanced asymmetry indices for paired regions (*A*_*r*_) as

(1) $$A_{r}=\frac{2|V_{R}-V_{L}|}{V_{R}+V_{L}}$$

for right and left regional volumes, *V*_*R*_ and *V*_*L*_. Unbalanced indices were generated by dividing *V*_*L*_ by *V*_*R*_.

The volumetry and asymmetry studies were carried out using the images acquired at 1.5 T. The findings were compared with published knowledge as a consistency check for the correctness of the segmentation approach.

### Comparison across field strengths

Where pairs of images acquired at 1.5 T and 3 T were available for individual subjects, the pair was rigidly registered and the unmasked label sets compared in the space of the 3 T image, using *JC*. A per-subject summary measure of agreement was obtained by calculating the mean *JC* across all 83 regions (*JC*_*m*_). Key results are also provided as Dice similarity coefficients [DSC, intersection divided by average label volume, (Dice, 1945)].

### Measuring precision by comparing independent segmentations

To derive a quantitative indicator of the precision of the segmentation of each target, we employed the following procedure. For each image in the ADNI set, we bisected the atlas set randomly into two subsets of 15 atlases each. From the pair of subsets, we generated a pair of independent segmentations using vote-rule decision fusion. The overall agreement between the unmasked segmentation pair was measured as the mean Jaccard coefficient across all 83 regions (*JC*_*m*_).

## Results and discussion

Segmentation results are available for download in NIfTI format as 3D label maps identifying 83 structures by spatial correspondence with the T1-weighted images as supplied by ADNI.^12^

### Quality of intracranial masks

The mean intracranial volume (ICV) obtained by measuring the volume of the intracranial mask (cf. Preprocessing) was 1.41 l (range 1.02–1.86 l, SD 0.143 l) on 1.5 T images. The images with the three largest (I72219, I40356, and I35499) and the three smallest (I63227, I82594, and I52799) ICVs were reviewed with the mask outline superimposed to search for visible under- and overestimations. All six masks were judged to adequately represent the intracranial volume after careful visual inspection.

In subjects for whom images had been acquired at both field strengths (*n* = 176), the measured ICV on 3 T was highly correlated with that of 1.5 T (Pearson's *r* = 0.976), giving smaller results on average than 1.5 T, but not significantly so (− 1%, range − 5*%*– + 10*%*, SD 2 percentage points, *p* = 0.32). Similar observations have previously been made, when ICV measurements were compared on pairs of brain images of subjects who had been scanned serially at different field strengths (Keihaninejad et al., 2010). Automatic methods showed a tendency to underestimate ICV on 3 T and overestimate ICV on 1.5 T images. For the most consistent automatic method described by Keihaninejad et al., the difference was 0.7%.

### Outlier analysis

Sixty regions in 42 subjects met the outlier criterion and were reviewed visually. Twelve subjects appeared twice in the list, two subjects appeared three times and one appeared four times. The regions that appeared most frequently in the outlier list were the temporal horn of the lateral ventricle (8 right, 6 left), the caudate nucleus (7 right, 5 left), and the subcallosal area (2 right, 4 left).

On visual review, the flagged regions appeared to be affected by white-matter disease in a large number of cases (WMD: 24; other flawed segmentations: 19, correct: 17). In 13 of the 19 problematic segmentations that were not WMD-related, the flaw appeared to be limited to the region in question. No further segmentation problems were detected in these cases, and the extent of over- or undersegmentation was deemed to be mild or moderate (scoring 3 or 4 on the visual analog scale described in Statistical and visual analysis section). In the remaining six regions, more general problems were seen and the relevant four cases (I64189, I38944, I67210, and I91126) were excluded from further analysis (MR acquisition problems leading to lack of GM/WM contrast: four regions in three images; motion artifact: two regions in one image).

WMD is a highly prevalent feature in the subjects of this cohort, frequently leading to overestimation of the caudate nuclei and the insula regions. The gray-matter portion of labels of other cortical regions often included white-matter regions that had been mistaken for gray matter by the tissue classification. Subcortical regions other than the caudate nuclei appeared largely unaffected on visual review. We determined for each image an index (cf. Quantifying white matter disease section) that signals WMD load. This index correlates well with the visual appearance of distortion (cf. Measuring WMD using white-matter hypointensities index). It is provided with the label images as part of the metadata.

The raw MAPER-based label for the lateral ventricle is frequently overestimated, incorrectly including hypointense portions of white matter. We dealt with this issue by masking this region pair with the binary CSF label generated by FAST.

While MAPER is robust in the majority of cases, the limitations of automatic segmentation (and, indeed, manual segmentation) need to be considered in subjects whose anatomical configuration is severely abnormal and in those who show texture abnormalities such as white matter disease.

### Measuring WMD using the white-matter hypointensities index

**Fig. 3:**
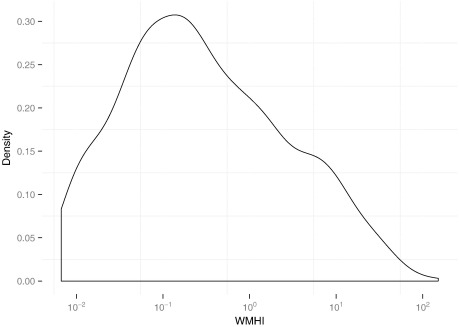
Density plot of white-matter hypointensities index (WMHI) on 996 images.

The WMHI ranged from 0 (seen in 135/996 images) to 151, with the distribution strongly skewed towards 0. The distribution is best visualized using a log scale as shown in Fig. 3.

**Fig. 4:**
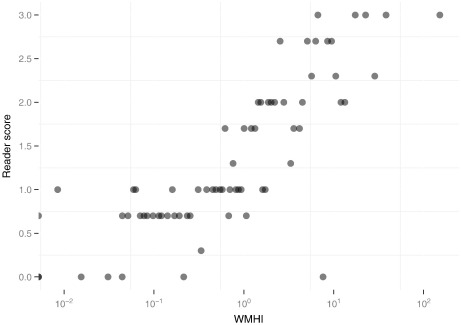
Scatter plot of white-matter hypointensities index (WMHI, log-scaled axis) versus the Wahlund score assigned by the rater on 70 images. WMHI scores of zero are shown as half disks on the left edge of the plot.

Fig. 4 plots the rater score against WMHI. The measures are strongly correlated (Kendall rank correlation coefficient 0.71, significant at the limit of numeric precision), although there is some overlap of WMHI between adjacent score groups. One image (I79803) received a visual score of 0, although it scored high on WMHI (7.59). Review of the MR image along with the intermediate Image *A* showing hypointensities showed an artefactual step change of intensity in the MR along the vertical axis, which resulted in a hypointense white matter region in the brainstem. No other white matter regions where highlighted or visibly affected by white matter disease.

The WMHI is intended to alert users to possible WMD-related oversegmentation when susceptible region labels are used for analysis. Such regions include the lateral ventricles before CSF-masking, the caudate nucleus and the gray-matter masked cortical regions, especially the insula. The WMHI has some value as a metadatum indicating the reliability of the segmentation. With a view to the caudate nucleus, however, its value is limited due to the way the index is generated: hypointensities adjacent to the caudate nucleus tend to be included in the generated caudate label, in which case they are not identified as WMD. Thus, it is possible for a caudate nucleus to be oversegmented due to white matter disease, even when the WMHI is zero. Random visual reviews have revealed one image where this appears to be the case (I63489). In future work, we will seek to address the issue of WMD-related oversegmentation in a principled fashion by identifying affected subjects and regions a priori and counteracting the distorting effects at the registration step. We will also search for better criteria to indicate the overall veracity of the generated segmentations.

### Volumetric analysis

#### Normalization

To reduce interindividual variation of region volumes, various measures have been proposed for normalization (Free et al., 1995). In particular, normalization of brain volume by intracranial volume was found to substantially reduce variation, and to remove gender-related differences (Whitwell et al., 2001). We found in previous work a correlation between hippocampal volume and ICV (Hammers et al., 2007), and this was confirmed in the present data (Pearson's *r* = 0.56 for the sum of both hippocampi in 1.5 T images of healthy subjects). Normalization by ICV also eliminates inaccuracies arising from problems with the phantom scaling, which have been reported for a subset of ADNI cases (Clarkson et al., 2009).

**Fig. 5:**
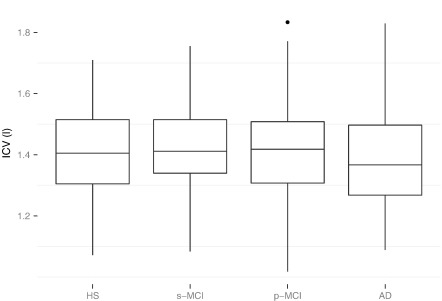
Comparison of intracranial volumes by diagnosis groups, 1.5 T images. Center line shows median, boxes capture 25%–75% quantile range, whiskers indicate 1.5 interquartile range, dots denote outliers. ICV unit is l.

Our ICV measurements were stable across the diagnostic groups (cf. Fig. 5). Based on a two one-sided tests (TOST) procedure (Schuirmann, 1987), the null hypothesis of non-equivalence can be rejected for all paired comparisons of diagnosis groups, except (s-MCI, AD) where *p* = 0.056 (*α* = 0.05; *ε* = 0.05*μ*). In the following, individual region sizes are expressed as a fraction of ICV, scaled by an arbitrary factor of 10^4^.

**Fig. 6:**
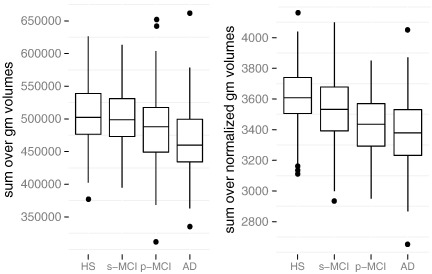
Summed volume of gray matter portions of labeled regions, comparison by diagnosis groups, 1.5 T images. Left: absolute volumes in mm^3^, right: normalized by ICV and scaled (arbitrarily) by 10^4^.

The benefit of ICV normalization can be seen in group comparisons by diagnosis: the absolute total gray matter volume differs between groups, but the distinction is comparatively weak (cf. Fig. 6, left panel). The right panel shows total gray matter volume with normalization, which results in larger group differences.

#### Aggregated regional analysis

**Fig. 7:**
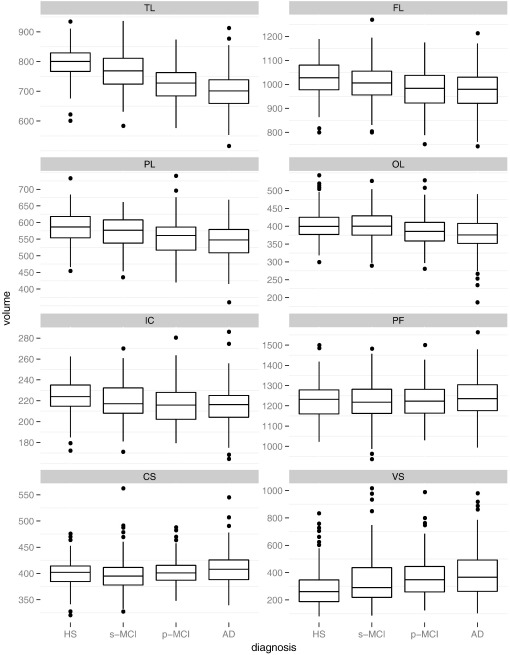
Summed label volumes per superregion, normalized and scaled by 10^4^. Gray matter masking applied where appropriate (all except CS, PF and VS). TL: temporal lobe, FL: frontal lobe, PL: parietal lobe, OL: occipital lobe, IC: insula and corpus callosum, PF: posterior fossa, CS: central structures, VS: ventricular system.

For Fig. 7, volume results for individual regions have been aggregated into six superregions. The plots indicate that the temporal lobe is most distinctly different between diagnostic groups. Differences in the medians are also substantial for the ventricle regions, but the variance is greater in all groups, resulting in larger overlaps.

#### Individual regional analysis

The analysis of individual regional volumes reveals a pattern of increasing atrophy from the HS group via s-MCI and p-MCI to the AD group. Table 3 shows this for the 14 regions where the AD–HS difference is largest. An extended version of the table that includes all regions is provided as supplemental material. Most of the results match our expectations: ventricles are enlarged, especially the temporal horns; hippocampi are smaller, notably also when comparing HS with s-MCI (9% either side). The amygdala, the middle and inferior temporal gyrus and the fusiform gyrus are reduced in size, adding to the evidence that temporal lobe regions beyond the hippocampus are affected by the disease process. The amygdala is functionally connected with and spatially adjacent to the hippocampus, and its involvement in AD is well known from histopathology (Kromer Vogt et al., 1990; Scott et al., 1991) and imaging research (Cuénod et al., 1993; Jack et al., 1997; Lehericy et al., 1994). Other temporal lobe structures, notably the fusiform gyrus, the parahippocampal gyri, and the middle and inferior temporal gyri also have previously been found to be significantly affected (Chan et al., 2001).

In recent imaging studies, thalamic volumes have been found to be reduced in Alzheimer's disease (Cherubini et al., 2010; Zarei et al., 2010), in line with earlier post-mortem observations (Braak and Braak, 1991). de Jong et al. (2008) found reduced sizes of both putamen and thalamus. Our results confirm lower volumes of the thalamus, even when comparing the HS and s-MCI groups (5% either side, highly significant). For the putamen, the same comparison was marginally significant, while the difference between HS and AD was not. This finding may indicate a limitation of accuracy of the putamen segmentation in subjects with more advanced disease.

**Fig. 8:**
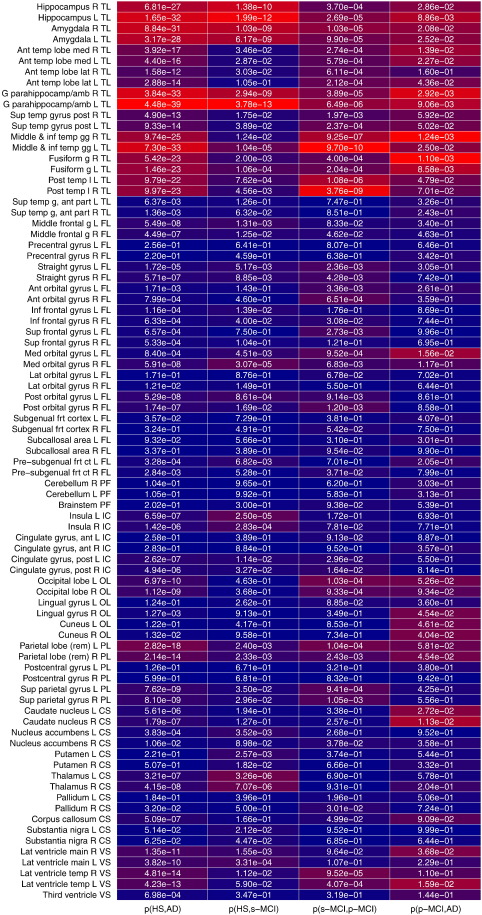
Heatmap showing per-region results of unpaired two-tailed t-tests between selected pairings of diagnosis groups. P-values are mapped to colors so that the “most significant” results for each column are highlighted in red. Each column is color-scaled independently. R: right, L: left, ant: anterior, amb: ambiens, temp: temporal, med: medial, lat: lateral, sup: superior, post: posterior, inf: inferior, g: gyrus, gg: gyri, l: lobe, frt: frontal, rem: remainder; superregion abbreviations as in Fig. 7.

The heatmap in Fig. 8 indicates for each region and selected pairs of diagnostic categories the extent to which the measured volume can serve to distinguish the diagnosis groups. Red color indicates the “most significant” results in each column. Please note that p-values in this context are not used for the usual purpose of hypothesis testing, but for comparing regions; therefore we did not employ alpha thresholding or attempt correction for multiple comparisons. Regions in the mesial temporal lobe (hippocampus, amygdala, and parahippocampal gyri) are particularly prominent, along with the temporal horn of the lateral ventricle and the posterior temporal lobe. Outside of the temporal lobe, large posterior cortical regions (parietal lobe, occipital lobe) are highlighted. These observations align well with previously described AD patterns, specifically a posterior-to-anterior gradient in atrophy (Likeman et al., 2005).

#### Asymmetry

Generally, AD atrophy is described as a disseminated process with no lateral predilection. Regional counts of plaques and tangles in pathological specimens showed larger variability within one and the same region than between left and right counterparts (Janota and Mountjoy, 1988; Moossy et al., June, 1989; Wilcock and Esiri, 1987). Imaging studies comparing AD with other entities found that asymmetry indices may be a useful tool for differential diagnosis, as asymmetry of various regions frequently attends clinically similar conditions, specifically frontotemporal lobar degeneration (Barnes et al., 2006; Boccardi et al., 2003; Horínek et al., 2007; Likeman et al., 2005) and argyrophilic grain disease (Adachi et al., 2010). As a differential diagnostic criterion, asymmetry thus speaks against AD according to these studies.

For the hippocampus, a physiological right-larger-than-left asymmetry in healthy adults is well established [e.g. Pedraza et al., (2004)], but studies focussing on hippocampal asymmetry in AD have yielded varying results. Small lateral differences in atrophy rates between AD patients and controls were found by Barnes et al. (2005). Shi et al. (2007), focussing on shape characteristics rather than volume, also found small differences between AD and controls in the atrophy pattern. A metastudy on hippocampal volume found that right hippocampal volume was larger than left in all groups studied (AD, MCI and controls), with AD subjects showing smaller effect sizes due to larger variation (Shi et al., 2009). Similarly, Barber et al. (2001) report a loss of hippocampal asymmetry in AD patients versus controls. An increase in hippocampal asymmetry as a function of cognitive decline was seen in one study (Wolf et al., 2001).

In the present study, results for the hippocampal left/right volume ratio have a wide distribution. We therefore choose to report the median and the median absolute deviation (MD), which are more robust measures of central tendency and dispersion than means and standard deviations. For healthy subjects, we found the previously reported pattern of left<right hippocampal asymmetry (median L/R volume ratio 0.93; MD 0.073). In AD, the median of the volume ratio appears to be somewhat reduced (0.90; MD 0.12), but the difference is not significant. The balanced asymmetry index *A*_*r*_ is higher in AD than in healthy subjects (0.12 versus 0.08), and this difference is significant (*p* = 1.3 × 10^− 5^).

Few studies have looked at asymmetry in AD beyond the hippocampus. The amygdala has been studied by Whitwell et al. (2005), who found a significant increase of asymmetry in AD patients. Thompson et al. (1998) found asymmetries in the Sylvian fissure in normal subjects, and these were significantly accentuated in AD.

**Fig. 9:**
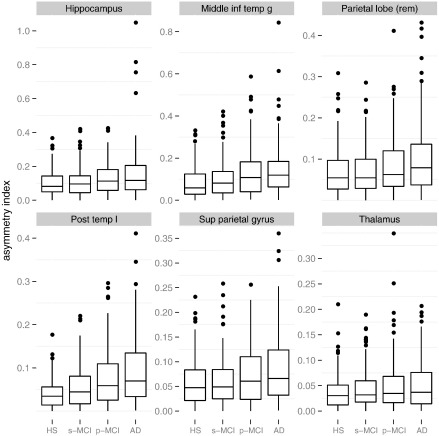
Boxplot showing group differences of asymmetry index for selected regions. The difference between AD and HS is highly significant for each of the regions shown (*p* < 10^− 4^); the difference between s-MCI and p-MCI is significant (*p* < 0.05) for the shown regions except thalamus and parietal lobe. Cf. Fig. 8 for abbreviations.

We note that *A*_*r*_ is particularly large in AD compared to HS when considering large regions (posterior temporal lobe, HS: 0.041, AD: 0.092, *p* = 1.3 × 10^− 15^, parietal lobe, HS: 0.068, AD: 0.100, *p* at limit of precision, cf. Fig. 9). This is an area for future exploration.

### Consistency across field strengths

High levels of agreement were seen when comparing the segmentation obtained on a 1.5 T image with that obtained on the same subject's 3 T image. The aggregate measure across all structures showed little variation between subjects (*JC*_*m*_ 0.802 ± 0.0146, range 0.749–0.829; corresponding to a DSC of 0.890). Results were equivalent for all four disease conditions as shown by a TOST procedure (*p* < 1e-17 for *α* = 0.05 and *ε* = 0.04). In previous work, we used the *JC*_*m*_ measure to assess MAPER with reference to manual segmentation in normal adults (Heckemann et al., 2010), obtaining a mean of 0.691 (DSC 0.817). The fact that the MAPER method produces consistent results across field strengths indicates high precision and corroborates our previous findings showing the accuracy of the method.

Between individual regions, we note large differences in the standard deviation of the Jaccard coefficient (*JC*_*σ*_). This standard deviation, here expressed as a percentage of the mean, ranges from 1.4% (brainstem) to 17.7% (left pallidum). Labels of regions that are well-defined by gray-scale intensity gradients in the T1 image are particularly consistent across field strengths. *JC*_*σ*_ for, e.g., the frontal horn and central part of the lateral ventricle is 3.0% (either side). For the precentral gyrus, which is a cortical region of average size within our set, *JC*_*σ*_ is 2.3% (left) and 2.5% (right). Small regions with weakly defined boundaries are naturally difficult to segment, both manually and by automatic procedures based on manual input. In the present results, this is reflected in large standard deviation values for the pallidum (*JC*_*σ*_ left: 17.7%, right: 14.3%) and the nucleus accumbens (left: 12.9%, right: 11.0%).

### Precision based on atlas subsets

There was strong agreement between independent atlas-subset based segmentations of the target images (*JC*_*m*_ was 0.800 ± 0.0092, range 0.771–0.819, corresponding to a DSC of 0.889). Three outliers were seen out of 996, one of each in the HS (I64867, *JC*_*m*_ 0.765), s-MCI (I69360, *JC*_*m*_ 0.718) and p-MCI (I97327, *JC*_*m*_ 0.755) groups. On visual review of the corresponding segmentations, no substantial mislabelling was seen.

### Future improvements

The choice of the segmentation approach employed in this work was guided by multiple considerations and represents a compromise that can be improved upon in future work. An important factor was proved robustness, as it helped to ensure that all segmentation results are traceable to a single procedure, and helped to avoid any individualized modification of the output data. As a consequence, a variety of recently published algorithmic developments have not been considered and may lead to even more accurate and detailed segmentations, once their robustness has been demonstrated. Promising developments are taking place in several areas. Registration algorithms are becoming more accurate and efficient as shown by Lötjönen et al. (2010) (albeit only for image pairs of identical provenance). The dependence on expensive expert input may in the future be reduced thanks to algorithms that uncover latent atlases (Riklin-Raviv et al., 2010). Procedures that select or weight atlas images from the outset (Aljabar et al., 2009) or at the segmentation combining stage (Artaechevarria et al., 2009) may yield more accurate results, especially when heterogeneous repositories are used as atlas data. Algorithms that revisit the image data after segmentation in order to refine the result are showing strong promise (Wolz et al., 2010b; van der Lijn et al., 2008).

A further compromise was made with regard to the choice of the atlas data. We settled on a set that has been segmented in high detail and with strong validation (Hammers et al., 2003), although it is based on young adults and is therefore demographically dissimilar from the ADNI target images. Work by Wolz et al. (2010a) shows for individual regions that the LEAP approach – propagating atlas labels indirectly via intermediate images – can yield improved results on such dissimilar targets. The question of how LEAP or a similar approach can be adapted for multiple regions is another promising research avenue.

## Conclusion

In this work we present a repository of label data on healthy elderly subjects and patients with mild cognitive impairment or Alzheimer's disease. We offer segmentations of 996 screening and baseline images in 816 subjects. The data are publicly available as an accompaniment to the MRI data supplied by the ADNI project. We validated the segmentation results and presented results of statistical analysis that are congruent with established knowledge about atrophy progression in AD.

We are committed to maintaining and enhancing the repository with findings from future research. In particular, we envisage developing further indices of accuracy and adding them as metadata. As improvements to the segmentation algorithm are developed and validated, we are planning to add updated segmentations, using versioning to ensure that the original set described here remains available for reference. If members of the community should express an interest in segmentations of follow-up scans, such data will also be added.

For researchers working with ADNI data, the repository will provide reliable information on a large number of anatomical regions. We envisage that the segmentations be used to search for novel imaging biomarkers of Alzheimer's disease and progressive mild cognitive impairment, using regional volume, shape, and texture information that can be derived. Our data will also enable region-based analysis of the functional imaging data acquired using positron-emission tomography, and of the connectivity data acquired using diffusion tensor imaging in the respective subsets.

## Figures and Tables

**Table 1:** Numbers of subjects in each group.

	HS	s-MCI	p-MCI	AD	Total
Female	101	74	64	88	327
Male	110	144	99	97	450
Total	211	218	163	185	777

**Table 2:** MAPER steps for generating an individual segmentation. Sim: similarity measure, mstprob: multi-spectral tissue probability map, CC: cross correlation, NMI: normalized mutual information. Numbers indicate the control point spacing in millimeters.

	Type	Level	Image data	Sim	Toolkit	Tool
1	Global	Rigid	mstprob	CC	IRTK	rreg
2	Global	Affine	mstprob	CC	IRTK	areg
3	Coarse	Nonrigid 20	mstprob	CC	IRTK	hreg
4	Detailed	Nonrigid 10, 5, and 2.5	T1 signal	NMI	Nifty Reg	reg_f3d
5	Transform	Nonrigid 2.5	Atlas labels	n/a	Nifty Reg	reg_resample

**Table 3:** Regional volumes and volume differences. Column “HS vol” shows regional volume as a fraction of ICV, averaged across healthy subjects, scaled by 10^4^. Columns labeled “d()” show the volume difference compared to HS as a percentage of HSvol, except “d(pMCI, sMCI)”, which shows the volume difference between p-MCI and s-MCI as a percentage of the mean volume of the s-MCI group. The sort criterion is the modulus of the difference between AD and HS. Only the 14 regions ranking highest on the sort criterion are shown. “Code” is the numerical identifier for the region used in the label maps.

Code	Region	HS vol	d(sMCI, HS)	d(pMCI, HS)	d(pMCI, sMCI)	d(AD, HS)
47	Lat ventricle temp horn R	5.3	10	32	21	43
45	Lat ventricle main R	125.2	17	27	8	40
46	Lat ventricle main L	138.1	21	31	9	39
48	Lat ventricle temp horn L	4.8	6	21	14	34
2	Hippocampus L	13.3	− 9	− 15	− 7	− 20
3	Amygdala R	9.5	− 8	− 15	− 7	− 19
10	G parahippocamp/amb L	24.3	− 9	− 15	− 7	− 19
1	Hippocampus R	14.4	− 9	− 14	− 6	− 18
4	Amygdala L	9.9	− 8	− 14	− 6	− 18
9	G parahippocamp/amb R	23.4	− 7	− 13	− 6	− 18
14	Middle and inf temp gg L	59.9	− 5	− 14	− 9	− 17
15	Fusiform g R	20.4	− 5	− 11	− 6	− 16
16	Fusiform g L	21.4	− 6	− 12	− 6	− 16
13	Middle and inf temp gg R	62.3	− 3	− 10	− 7	− 14

## Appendix A Supplementary data

Regional volumes and volume differences. Cf. Table 3 for abbreviations and column labels.

## Footnotes

3 http://www.med.harvard.edu/AANLIB.

4 http://www.braincolor.org/protocols.

5 http://www.doc.ic.ac.uk/~dr/software.

6 http://sourceforge.net/projects/niftyreg.

7 http://www.picsl.upenn.edu/ANTS.

8 http://www.nitrc.org/projects/art.

9 ADNI was launched in 2003 by the National Institute on Aging (NIA), the National Institute of Biomedical Imaging and Bioengineering (NIBIB), the Food and Drug Administration (FDA), private pharmaceutical companies and non-profit organizations, as a $60 million, 5-year public–private partnership. The Principal Investigator of this initiative is Michael W. Weiner, M.D., VA Medical Center and University of California San Francisco. ADNI is the result of efforts of many co-investigators from a broad range of academic institutions and private corporations, and subjects have been recruited from over 50 sites across the U.S. and Canada. The initial goal of ADNI was to recruit 800 adults, ages 55 to 90, to participate in the research — approximately 200 cognitively normal older individuals to be followed for 3 years, 400 people with MCI to be followed for 3 years, and 200 people with early AD to be followed for 2 years.

10 http://www.loni.ucla.edu/twiki/bin/view/ADNI/ADNIMRICore.

11 Originally, we had sampled 21 evenly from the ranked list. Based on the review of this original set, we decided to increase the sample size. The trisection approach allowed us to add to the existing sample, while maintaining even spacing within the parts and emphasizing the upper WMHI value range, where we expected the findings to be most relevant.

12 ADNI users can download the label maps from the image database (https://ida.loni.ucla.edu) via the “Advanced Search” feature by selecting “Post-processed” and entering “MAPER*” in the field “Series Description”.
